# An Integrated Workflow for Three-Dimensional Visualization of Human Skeletal Muscle Stem Cell Nuclei

**DOI:** 10.21769/BioProtoc.5281

**Published:** 2025-04-20

**Authors:** Jeremy R. Pearson, Noraida Martinez-Rivera, Irma Torres-Vasquez, Philip M. Gallagher, Eduardo Rosa-Molinar

**Affiliations:** 1 Microscopy and Analytical Imaging Research Resource Core Laboratory, University of Kansas, Lawrence, KS, USA; 2 Department of Pharmacology & Toxicology, University of Kansas, Lawrence, KS, USA; 3 Applied Physiology Laboratory and Osness Human Performance Laboratories, University of Kansas, Lawrence, KS, USA; 4 Department of Cell Biology & Physiology, and Neuroscience, Washington University Center for Cellular Imaging, Washington University School of Medicine, St Louis, MO, USA

**Keywords:** Satellite cells, Immunohistochemistry, Muscle regeneration, Fluorescent microscopy, Muscle damage, Myofiber cross-sectional area

## Abstract

Skeletal muscle–specific stem cells are responsible for regenerating damaged muscle tissue following strenuous physical activity. These muscle stem cells, also known as satellite cells (SCs), can activate, proliferate, and differentiate to form new skeletal muscle cells. SCs can be identified and visualized utilizing optical and electron microscopy techniques. However, studies identifying SCs using fluorescent imaging techniques vary significantly within their methodology and lack fundamental aspects of the guidelines for rigor and reproducibility that must be included within immunohistochemical studies. Therefore, a standardized method for identifying human skeletal muscle stem cells is warranted, which will improve the reproducibility of future studies investigating satellite activity. Additionally, although it has been suggested that SC shape can change after exercise, there are currently no methods for examining SC morphology. Thus, we present an integrated workflow for three-dimensional visualization of satellite cell nuclei, validated by the spatial context of the fluorescent labeling and multichannel signal overlap. Our protocol includes, from start to finish, post-biopsy extraction and embedding, tissue sectioning, immunofluorescence, imaging steps and acquisition, and three-dimensional data post-processing. Because of the depth volume generated from the confocal microscope z-stacks, this will allow future studies to investigate the morphology of SC nuclei and their activity, instead of traditionally observing them in two-dimensional space (x, y).

Key features

• Detailed instructions on post-biopsy extraction and embedding, tissue sectioning, immunofluorescence, imaging steps and acquisition, and three-dimensional data post-processing of muscle stem cells.

• Builds upon the validated method developed by Feng et al. [1], which was optimized for mouse tissue and fills critical gaps in existing literature.

• Allows qualitative and quantitative morphological assessment of muscle stem cell nuclei in three-dimensional space.

Graphical overview

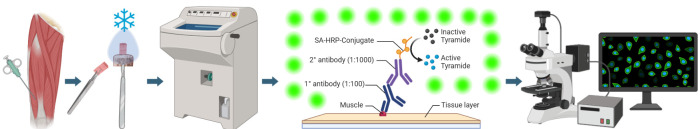

**Graphical overview of integrated workflow for three-dimensional visualization of human skeletal muscle stem cells.** After the percutaneous muscle biopsy, cut ~25–100 mg of the sample and arrange it according to the desired orientation → Mount sample for sectioning, embed in mounting medium, and freeze in liquid nitrogen-cooled isopentane → Using a cryostat, generate tissue cross-sections in an alternating collection method of 20 μm intervals and place on subbed glass slides → Fix and block sections before incubating in a cocktail of primary antibodies specific for satellite cell nuclei (anti-Pax7) and muscle membrane (anti-laminin) overnight. The following day, incubate sections in the appropriate secondary antibodies (Pax7: goat anti-mouse IgG1 biotin conjugated; laminin: goat anti-rabbit Alexa Fluor 488), apply signal amplification using streptavidin-horseradish peroxidase and tyramide 594 conjugate before counterstaining with DAPI, add mounting media, and coverslip → Using a confocal microscope, search for Pax7 signal, confirm overlap with DAPI adjacent to laminin labeling, apply appropriate laser channels, determine z-stack size, and acquire images in high-pixel-resolution format → For image post-processing, in the software’s three-dimensional viewer, modify individual channel histograms to optimize image quality and save as a TIFF.

## Background

**Figure 1.**
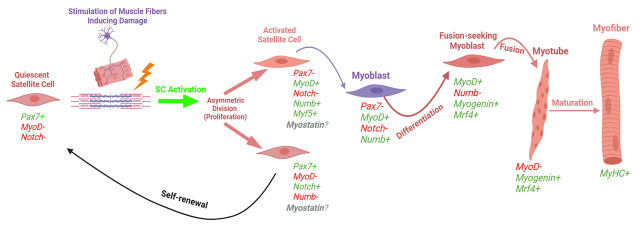
Myogenic lifespan of a satellite cell following exercise-induced muscle damage (adapted, modified, and used with permission from Vlasman [7]). Schematic representation of the two routes that satellite cells can take upon activation. Activated satellite cells can proliferate and then either commit to differentiation (expressing myogenin, MyoD, and MRF4) or self-renew back into a quiescent state (only expressing Pax7).

Intense physical activity can induce acute skeletal muscle damage that can persist for multiple days and even up to a few weeks [2]. In individuals with muscle-specific diseases such as sarcopenia, muscular dystrophy, or cachexia, this damage can be exacerbated due to impaired muscle regeneration mechanisms, often leading to progressive muscle wasting and functional decline [3]. Muscle stem cells, also referred to as satellite cells (SCs), are responsible for the repair of those injured fibers. Once damage occurs, SCs can activate, proliferate, and differentiate to assist in the recovery process. In addition to the differentiation process, SCs can self-renew, allowing for the quiescent SC pool to maintain its abundance. The path of the SC (i.e., differentiation, self-renewal, etc.) is contingent on myogenic transcription factors, which dictate the fate of the SC (Bellamy et al. [4]; Figure 1). These myogenic biomarkers can be visualized for identifying SC activity through fluorescent imaging. It is essential to study and understand these cells due to their crucial role following exercise and on skeletal muscle health and longevity; it has also been shown that SC exhaustion is linked to sarcopenia, which significantly increases the risk of mortality [5,6].

A fundamental area of skeletal muscle biology research has been identifying and monitoring the activity of satellite cells. One of the techniques for investigating SCs is the application of indirect immunofluorescence. This technique involves using a specific primary antibody that can bind to an epitope on the SC; then, with the help of a fluorophore-conjugated secondary antibody bound to the primary antibody, SCs can be visualized with a fluorescent microscope. For example, Pax7, a transcription factor highly expressed within the nuclei of SCs, has been suggested to be one of the most utilized primary antibodies for identifying SCs [1]. However, this is challenging, as SC nuclei comprise only 2%–7% of the total muscle-associated nuclei (e.g., myonuclei) in healthy adults and even less in elderly populations [8], making them hard to locate in small tissue samples.

This protocol was designed to address key methodological gaps in previous literature. The SC identification protocols within the literature are missing fundamental aspects of the guidelines for rigor and reproducibility that must be included within immunohistochemical studies [9]. For example, there is a high degree of variability in the reagents used (e.g., antibodies, buffers, use of signal amplification products, or dilutions), experimental instructions, and published images. Also, these methodologies lack essential components within the experimental procedures including positive and negative controls, imaging instructions, and quantification of images. This reduces the potential for other laboratories to generate reproducible data. Additionally, to our knowledge, all studies investigating human SCs have sectioned the muscle tissue between 5 and 12 μm, which is problematic because SC length can reach up to 15 μm [10–12]. Specifically, cutting relatively thin sections can increase the possibility of error when analyzing SC quantity, due to the chance for duplication counts of the same cell on different sections. Therefore, we provide an optimized, detailed, and comprehensive protocol for visualizing human skeletal muscle SC nuclei in three-dimensional space, enabling the identification and morphological analysis of quiescent SC nuclei. This protocol was designed to address the key methodological gaps and has the potential to offer multiple advantages, including the ability to identify morphological changes in SC nuclei at rest, after exercise or nutritional interventions, and in healthy vs. diseased states.

## Materials and reagents

**Biological materials**

1. Human skeletal muscle biopsy sample (male; 28 years old; 79 kg, 172 cm)

2. C2C12 (mouse myoblast) cell line (ATCC; CRL-1772) used as positive and negative controls

**Reagents**

1. Liquid nitrogen

2. Deionized water (diH_2_O)

3. Gum tragacanth (Sigma-Aldrich, catalog number: G1128)

4. 2-Methylbutane/isopentane 97.0+% (Thermo Fisher Scientific, catalog number: M0167500ML)

5. Paraformaldehyde (Sigma-Aldrich, catalog number: P6148-1KG)

6. Fisher optimal cutting temperature (OCT) compound (Thermo Fisher Scientific, catalog number: 23-730-571)

7. Antifade mounting media (VectaShield, Vector Laboratories, catalog number: H-1000)

8. 10× Phosphate buffered saline solution (PBS) (Thermo Fisher Scientific, catalog number: 70-011-044)

9. Saponin (Sigma-Aldrich, catalog number: 84510)

10. Sucrose (Thermo Fisher Scientific, catalog number: S5)

11. Hanks’ balanced salt solution (HBSS) 10× (Thermo Fisher Scientific, catalog number: 14185-052)

12. Dulbecco’s modified Eagle’s medium, high glucose (4.5 g/L) + L-glutamine without sodium pyruvate (Corning, Life Sciences, catalog number: 10-017-CV)

13. Gibco^TM^ fetal bovine serum (FBS), qualified (Thermo Fisher Scientific, catalog number: 26140079)

14. Penicillin-streptomycin (10,000 U/mL) (Thermo Fisher Scientific, catalog number: 151440122)

15. Normal goat serum (Jackson ImmunoResearch, catalog number: 005-001-121)

16. 30% hydrogen peroxide (H_2_O_2_) (Thermo Fisher Scientific, catalog number: H325-100)

17. 2.5% normal horse serum (NHS) (Jackson ImmunoResearch, catalog number: 008-000-001)

18. Acetone (fixative solution) (Thermo Fisher Scientific, catalog number: A16P-4)

19. 4',6-diamidino2-phenylindole (DAPI) (Thermo Fisher Scientific, catalog number: D1306)

20. Type F immersion liquid (Leica Microsystems, catalog number: 11513859)

21. Gelatin (Electron Microscopy Sciences, catalog number: 16562)

22. Chromium potassium sulfate (Sigma-Aldrich, catalog number: 243361)

23. Immunohistochemistry reagents (Table 1)

**Table 1.** Immunohistochemistry antibodies and reagent information

Antibody/reagent	Company	Catalog number	Reactivity	Host/isotype	Dilution/diluent	Storage
Pax7 (concentrate) monoclonal primary antibody **Caution:** We could not optimize this protocol with the supernatant (dilutions 1:10, 1:50, and 1:100) or a different Pax7 primary antibody (Rabbit anti-Pax7 polyclonal IgG; Thermo Fisher Scientific, cat no.: PA1-117)	Developmental Studies Hybridoma Bank	Pax7	Human	Mouse/IgG1	(1:100)/2.5% NHS	-20 °C
Biotinylated-SP goat anti-mouse IgG1 secondary antibody	Jackson ImmunoResearch	115-065-205	Mouse IgG1	Goat/IgG1	(1:1,000)/2.5% NHS	-20 °C
Laminin polyclonal primary antibody	Sigma-Aldrich	L9393	Human	Rabbit/IgG	(1:200)/2.5% NHS	-20 °C
Alexa Fluor 488 goat anti-rabbit IgG secondary antibody	Thermo Fisher Scientific	A-11034	Rabbit	Goat/IgG	(1:200)/PBS	4 °C
Alexa Fluor 594 tyramide	Thermo Fisher Scientific	B40957	HRP	N/A	(1:200)/PBS	4 °C
Streptavidin horseradish peroxidase (SA-HRP)	Thermo Fisher Scientific	S911	Biotin	N/A	(1:500)/PBS	-20 °C
Alexa Fluor 647 goat anti-mouse secondary antibody **Caution:** This secondary antibody was used with the supernatant primary antibody, which we could not optimize for this protocol	Thermo Fisher Scientific	A21236	Mouse	Goat/IgG	(1:200)/1× HBSS + 0.1% sucrose + 1% saponin	4 °C

*Note: Prepare on the day of the experiment.*

**Solutions**

1. 1× Phosphate buffered saline (PBS) (see Recipes)

2. Subbing solution for glass slides (see Recipes)

3. 2.5% Normal horse serum (blocking buffer) (see Recipes)

4. 3% hydrogen peroxide (see Recipes)

5. Ice-cold acetone fixative solution (see Recipes)

6. 10 mM 4',6-diamidino2-phenylindole (DAPI) (see Recipes)

**Recipes**

**1. 1× Phosphate buffered saline (PBS) (100 mL)**

**Table:** 

Reagent	Quantity/Volume
PBS 10×	10 mL
diH_2_O	900 mL

Prepare the day before. Store at 4 °C.

**2. Subbing solution for glass slides (1 L)**

**Table:** 

Reagent	Quantity/Volume
Gelatin	1 g
Chromium potassium sulfate	0.1 g
Hot distilled H_2_O	1 L

Mix well and let it cool before adding chromium potassium sulfate. Submerge slides and place them vertically on a dry paper towel. Stock can be stored at 4 °C; aliquot working volumes in a coupling jar.

**3. 2.5% normal horse serum (blocking buffer) (3 mL)**

**Table:** 

Reagent	Quantity/Volume
Normal horse serum	75 μL
1× PBS	2,925 μL

Prepare the day before. Store at 4 °C.

**4. 3% hydrogen peroxide (1 mL)**

**Table:** 

Reagent	Quantity/Volume
30% H_2_O_2_	30 μL
1× PBS	970 μL

Prepare the day before. Store at 4 °C.

**5. Ice-cold acetone fixative solution (1 mL)**

**Table:** 

Reagent	Quantity/Volume
Acetone	1 mL

Prepare on ice the day before. Store at -20 °C.

**6. 10 mM 4',6-diamidino2-phenylindole (DAPI) (50 mL)**

**Table:** 

Reagent	Quantity/Volume
14.3 mM DAPI	35 μL
diH_2_O	49.955 μL

Prepare the day before. Reconstitute with 2 mL of diH_2_O. Store at 4 °C; if protected from light, it is usable long-term.

**Laboratory supplies**

1. Petri dishes 100 mm Nunc (Thermo Fisher Scientific, catalog number: 263991)

2. Reynold wrap aluminum foil (Grainger, catalog number: 6CHG6)

3. Superfrost Plus slides (Thermo Fisher Scientific, catalog number: 12-550-15)

4. 22 × 50 mm No. 1.5 (0.16–0.19 mm) thick coverslip glass (Ted Pella, catalog number: 260154)

5. Ibidi μ-slide 8-well glass bottom (Ibidi, catalog number: 80827)

6. Kimwipes (KCWW, Kimberly-Clark, catalog number: 34120)

7. Gloves (VWR, catalog number: 82026-424)

## Equipment

1. Bergström needle (5 mm) composed of the outer cannula, inner trocar, and plunger (Pelomi Medical, Albertslund, Denmark, catalog number: 1226-005) with associated components: 200 μL pipette tip with ~15–18 mm cutoff, 30 cm extension tubing, and disposable 140 mL Monoject bulk syringe (Cardinal Health, catalog number: 8881114055)

2. Lab-grade stopper cork (size 00) for muscle mount (Thermo Fisher Scientific, catalog number: 07-782G)

3. Swann-Morton scalpel with blade size 11, sterile (Thermo Fisher Scientific, catalog number: 50-192-8439)

4. Styrofoam cryo dewar (for liquid nitrogen) (Agar Scientific, catalog number: AG81760-130)

5. Cryostat microtome (Leica Microsystems, model: CM1800)

6. HP35 ultra-microtome disposable blades at 34°/75 mm (Thermo Fisher Scientific, catalog number: 31-537-35)

*Note: We recommend that slides be coated with subbing solution before collecting samples.*

7. Humidifying slide chamber (6-slide clear staining tray with clear lid) (Sigma-Aldrich, catalog number: H6644-1EA)

8. Spectrafuge^TM^ 16M brushless laboratory microcentrifuge (Labnet, catalog number: C0160)

9. 0.5–10 μL mechanical pipette (Eppendorf, catalog number: 3123000020)

10. 20–200 μL mechanical pipette (Eppendorf, catalog number: 3123000055)

11. 100–1,000 μL mechanical pipette (Eppendorf, catalog number: 3123000063)

12. 500 μL plastic microcentrifuge tubes (Thermo Fisher Scientific, catalog number: AM12350)

13. 500 mL Pyrex round, wide-mouth media storage bottle with GLS 80 screw cap (Corning Life Sciences, catalog number: 1397-500)

14. Camel hair paint brush, fine tip (size 0.5–3) for sectioning (Grainger, catalog number: 39AL12)

15. Forceps, fine tip (Thermo Fisher Scientific, catalog number: 22-327379)

16. Clear nail polish for sealing slides (Thermo Fisher Scientific, catalog number: 50-949-071)

17. Rounded flat-end scoop spatula (Grainger, catalog number: 56HV79)

18. 4 °C refrigerator

19. Epifluorescent inverted microscope (Olympus, model: IX-81)

20. Laser scanning confocal upright microscope (Leica Microsystems, model: DM6-Q)

## Software and datasets

1. LAS X digital imaging software version 3.5.7.23225 (Leica Biosystems)

2. BioRender (https://www.biorender.com/). The following figures were created using BioRender: Graphical overview; Figure 1)

## Procedure

In this protocol, human skeletal muscle tissue is extracted, embedded, sectioned, and then processed to identify satellite cell nuclei in three-dimensional space using confocal microscopy.

**Figure 2.**
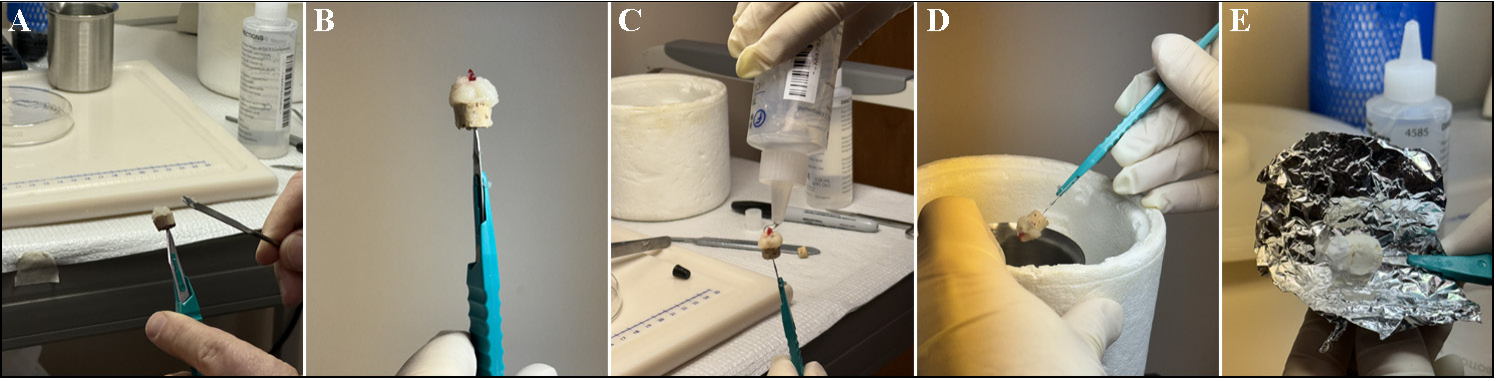
Embedding process of muscle tissue. (A) Prepare a cork mount by cutting a stopper cork in half through the center of the cork to make a platform for mounting media; attach the bottom of the cork to a #11 scalpel. When the tissue is ready to be mounted, layer and apply mounting medium and then use fine tweezers to split the mounting media in half to make room in the middle for the sample. (B) Use fine-tipped forceps and a spatula to carefully place the cut muscle sample (fibers running parallel) straight up (try to be precise) onto the cork like a cylinder. Use the spatula to gently work the mounting medium in to encapsulate the sample to close any gaps between the muscle and outside air. (C) Apply a cover layer of OCT to further protect the tissue from any outside air. (D, E) Once the isopentane is ready (crystals forming at the bottom), flip the sample upside down and immerse the cork to where the sample is fully submerged in the liquid nitrogen–cooled isopentane until OCT hardens over the sample (i.e., ~30 s). Detach the embedded tissue from the scalpel and place it in aluminum foil on a plastic Petri dish floating above liquid nitrogen for 60 min. This ensures non-rapid freezing and prevents freeze-fracture. Afterward, place it in an appropriate storage area until ready to section. The embedded tissue can be stored at -80 °C for long-term (2 years) or at -20 °C for short-term storage (6 months).

**A. Muscle biopsy embedding protocol** (see Figure 2) [13,14]

Approximately 150–300 mg of skeletal muscle tissue is extracted from the vastus lateralis via the modified Bergstrom percutaneous muscle biopsy technique with suction [15,16].

*Note: Perform steps A1–A4 20 min before biopsy.*

1. Prepare a liquid nitrogen container (e.g., 5’’ circular Styrofoam cryo dewar).

2. Prepare the OCT embedding solution and mounting medium.

a. In a 20 mL beaker, add ~100–150 μg of gum tragacanth and 3–4 small drops of water, mixing with a laboratory spatula.

b. Add 1:1 OCT compound and mix to make a thick paste.

3. Prepare 2-methylbutane (isopentane): pour 10 mL of isopentane into a small (e.g., 125 mL) stainless-steel container.

4. Prepare cork mount:

a. Cut a stopper cork (size zero) in half. With the bigger half, place a Swann-Morton scalpel through the center of the cork to make a platform for mounting media. Attach the bottom of the cork to a #11 scalpel.

b. When tissue is ready to be mounted, layer and apply mounting medium, and then use fine tweezers to split mounting media in half to make room in the middle for the sample.

5. Following sample extraction from the inner trocar of the Bergström needle, place the muscle on a sterilized Petri dish, remove visible connective tissue (e.g., blood, fat, fascia) with a scalpel, and rinse with 1× PBS if the muscle is coated in excessive blood.

6. Using a sterile scalpel, cut approximately half the sample depending on the size and desired orientation; it is important to have intact fibers for immunohistochemistry (~0.5–0.75 cm in length and 25–100 mg of total tissue).

7. Place a stainless-steel container of isopentane into the Styrofoam container with liquid nitrogen and wait for the isopentane to begin crystallizing.

8. For processing and analyzing cross-sections, use a stereomicroscope with LED pipe lights and tweezers to examine the orientation of the muscle sample.

9. Use fine-tipped forceps and a spatula to carefully place the cut muscle sample (fibers running parallel) straight up (try to be precise) onto the cork like a cylinder. Using the spatula, gently work the mounting medium in to encapsulate the sample and close any gaps between the muscle and outside air.

*Note: If the muscle sample is exposed in a certain area to outside air, this will cause freeze-damage to the muscle tissue.*

10. Apply a cover layer of OCT to further protect the tissue from any outside air.

11. Once the isopentane is ready, flip the sample upside down and immerse the cork so that the sample is fully submerged in the liquid nitrogen-cooled isopentane until OCT hardens over the sample (i.e., ~30 s).

*Note: If the isopentane is ready, after a few seconds in liquid nitrogen, it should start solidifying, and white solids should form on the bottom of the stainless-steel container.*

12. Detach the embedded tissue from the scalpel and place it in aluminum foil on a plastic Petri dish floating above liquid nitrogen for 60 min. This ensures non-rapid freezing and prevents freeze-fracture. After that, place it in an appropriate storage area until ready to section. The embedded tissue can be stored at -80 °C for long-term storage (2 years) or at -20 °C for short-term storage (6 months).

**B. Sectioning protocol using cryostat**

1. Set cryostat temperature to -23 °C and stage knife cutting angle to 4°.

*Note: Other lab protocols have recommended a cutting angle set between 8° and 10°.*

2. Label the Superfrost slides by including subject/sample number, slide number, primary and secondary antibodies and stains (e.g., DAPI) applied, date of experiment, and initials of the investigator.

3. Once cooled, obtain the sample from the freezer or fridge and quickly transfer to the cryostat chamber, making sure the sample stays frozen.

4. Apply a layer of OCT to the chuck stub (i.e., specimen holder), place the sample flat and perpendicular to the knife into the OCT, and use a cold spray to quickly spray around where the new OCT was added to allow solidification.

*Note: Other lab protocols have recommended a 1 h incubation so that the mount acclimates to the temperature and prevents the cork from tearing off the chuck while sectioning.*

5. Mount the chuck stub into the holder and align it to the knife stage so that the sample is perpendicular to the blade; input and orient the appropriate microtome blade.

6. Begin facing the block. Once the tissue is visualized in the block, section the sample in an alternating method of 20 μm intervals (i.e., collect→ trash → collect→ trash) and collect the cryo-sections on frosted, subbed Superfrost slides with approximately three tissue sections per slide. Make sure sections are centered away from the edges of the slide.

*Note: We recommend alternate sectioning to control for duplicate sampling. Also, if the goal is to quantify, this way you can ensure the analysis will not be of the same cell. If sections are not flattening correctly, remove the anti-roll plate and, with one hand, use a small camel hair paint brush (e.g., size 2) to pull the section as it is being cut while your other hand controls the microtome wheel.*

7. Dry the sections at room temperature (RT) for 30 min and proceed immediately to the fixation and staining protocol. If using a PAP pen, circle samples to be stained.

*Note: Although drying the sections in the freezer overnight or for long-term storage at -80 °C or -20 °C for later use is acceptable, this increases background autofluorescence. For best results, use immediately.*

**C. Immunofluorescence protocol** (adopted and modified from Fry et al. [17])

For our example protocol, we label four Superfrost slides with three tissue sections on each slide. Slides 1 and 2 were labeled with Pax7 + laminin + DAPI stain, slide 3 was labeled with Pax7 + DAPI stain (external control for laminin), and slide 4 was labeled with laminin + DAPI stain (external control for Pax7). We recommend 75 μL of working buffer per tissue section to be fully covered on a subbed, flat glass slide. A PAP pen can be used to prevent the reagent from running off the tissue sections and keep them covered; however, we do not use a PAP pen. Other labs have used a Coplin jar for submerging sections; however, this requires significantly higher amounts of reagents and buffers.

**Day 1:**

1. Place slides in a plastic humidity chamber with a moist paper towel that is soaked flat inside the reservoir to keep humid, so the buffer/reagents do not evaporate.

*Note: Using the humidity chamber for this protocol makes it very efficient to quickly remove and replace buffer/reagent (discard/dump into paper towel).*

2. Fix tissue sections in ice-cold acetone for 3 min.

a. Place aliquoted acetone on ice, pipette 75 μL (or appropriate volume) to each section for each slide, and quickly remove acetone after 3 min.

*Note: The longer the incubation in acetone, the higher the chance of autofluorescence. Additionally, methanol is not recommended as a fixative solution due to its high autofluorescence. However, we have previously used 2%–4% formaldehyde.*

3. Using a pipette, wash tissue sections three times × 3 min with 1× PBS (aliquot the 1× PBS into a small beaker for the protocol to avoid contamination) to remove the fixative solution.

4. Block endogenous peroxidase.

a. Add 75 μL (or appropriate volume) of 3% H_2_O_2_ in 1× PBS for 7 min at RT.

*Note: Bubbles forming on the section indicate that the peroxide is working; a lack of bubbling will not affect the staining outcome but indicates poor tissue quality.*

5. Using a pipette, wash tissue sections three times × 3 min with 1× PBS to remove the H_2_O_2_ solution.

6. Total protein blocking:

a. Add 75 μL (or appropriate volume) of 2.5% normal horse serum (i.e., blocking buffer) to each section and incubate for 60 min at RT.

*Note: We previously used 0.1%–0.2% Triton X-100 in this blocking step as a permeabilization agent and the results were suboptimal. Since no difference was observed, we opted to maintain the integrity of the cell membrane and use the blocking bluffer without any detergent.*

b. Begin preparing the primary antibodies: 2 slides/6 tissue sections of Pax7+laminin (cocktail); 1 slide/3 tissue sections of Pax7 only; 1 slide/3 tissue sections of laminin only.

**Figure 3.**
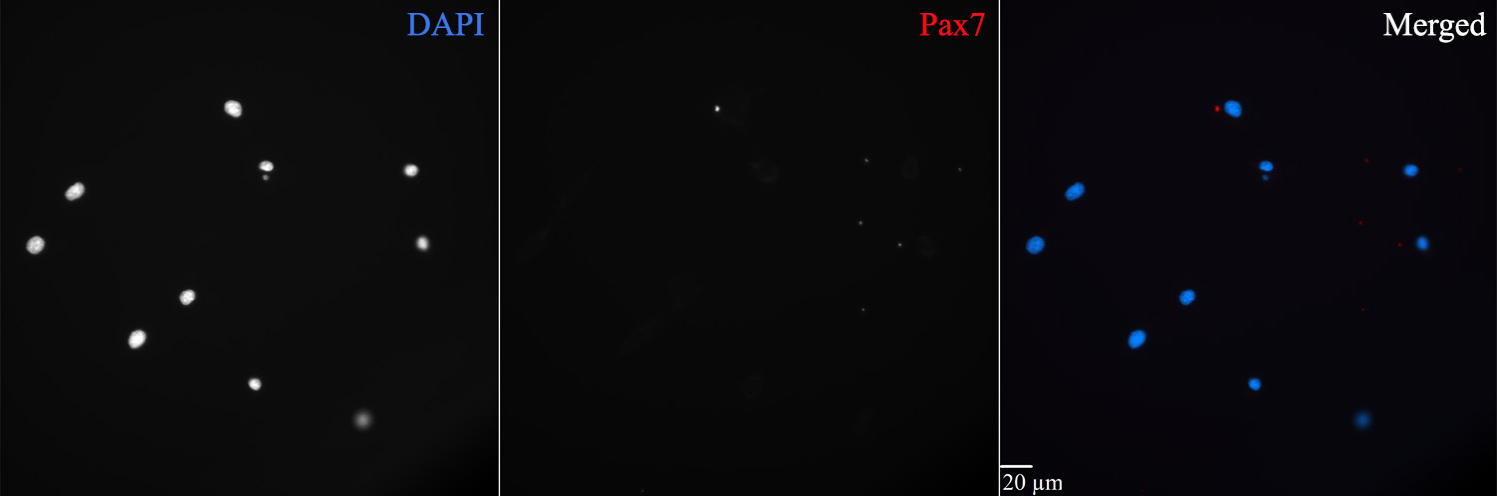
Immunohistochemical negative control for validating the specificity of Pax7 labeling. The C2C12 (ATCC CRL-1772) mouse myoblast cell line incubated in sp2/0-ag14 conditioned serum media was used as a negative control for Pax7 identification. The sp2/0-ag14 serum replaced the traditional horse serum, which induces differentiation and expression of Pax7. This figure plate shows a very weak nonspecific background signal but not Pax7 positive labeling. C2C12 cells were thawed at passage 13 (P13) and cultured for 5 days in Dulbecco’s modified Eagle’s medium + 1% penicillin-streptomycin + 10% heat-inactivated FBS at 37 °C in an incubator with 5% CO_2_. Cells were then passaged and incubated in the same culture media but with sp2/0-ag14 conditioned sera for 3 days. The immunohistochemical protocol was run at cell passage 14 (P14) in an 8-well Ibidi μ-slide with a 200 μL working volume. Briefly, cells were fixed in 2% formaldehyde for 10 min, blocked in 3% hydrogen peroxide for 7 min to quench endogenous peroxidase activity, blocked in 3% normal goat serum diluted in 1× HBSS+0.1% sucrose+1.0% saponin for 20 min, and incubated overnight in a mouse monoclonal anti-Pax7 IgG1 primary antibody (dilution 1:100). The next day, cells were incubated in goat anti-mouse IgG1 biotin-SP-conjugated secondary antibody (dilution 1:1,000) for 60 min, incubated in streptavidin horseradish peroxidase conjugate for 60 min, amplified with Alexa Fluor 594 tyramide conjugate (dilution 1:200) for 20 min, and counterstained with 10 mM DAPI for 30 min. VectaShield antifade mounting media was added, and plates were stored at 4°C until imaging. Images were acquired using an Olympus IX-81 epifluorescent inverted microscope with the Olympus UPlanSApo 40×/0.95 NA long-working-distance objective and SlideBook 6.0 software. Scale bar = 20 μm.

**Figure 4.**
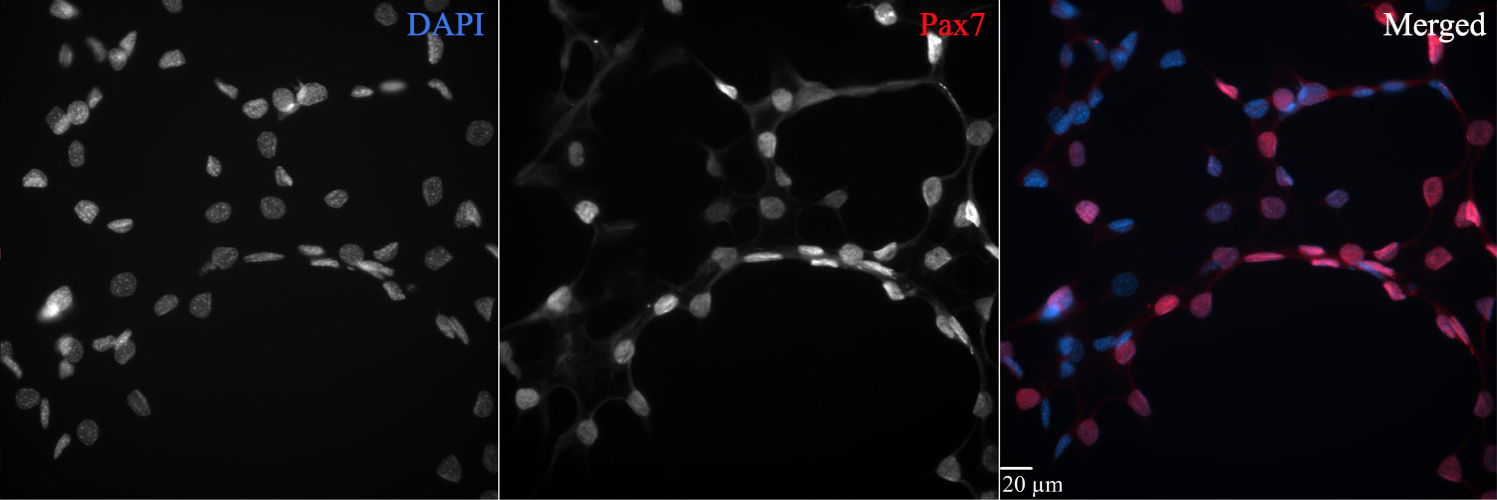
Immunohistochemical positive control for validating the specificity of Pax7 labeling. The C2C12 (ATCC CRL-1772) mouse myoblast cell line incubated in differentiation media containing horse serum was used as a positive control for Pax7 identification. Horse serum induces differentiation and expression of Pax7 when applied for more than two days. This figure plate shows a strong overlapping signal with DAPI (merged image), indicating similar signal intensities in both the muscle tissue samples and C2C12 cells. C2C12 cells were thawed at passage 12 (P12) and cultured for two weeks in Dulbecco’s modified Eagle’s medium + 1% penicillin-streptomycin + 10% heat-inactivated FBS at 37 °C in an incubator with 5% CO_2_. Cells were then passaged and incubated in differentiation culture media replacing FBS with horse serum for 3 days. The immunohistochemical protocol was run at cell passage 13 (P13) in an 8-well Ibidi μ-slide with a 200 μL working volume. Briefly, cells were fixed in 2% formaldehyde for 10 min, blocked in 3% hydrogen peroxide for 7 min to quench endogenous peroxidase activity, blocked in 3% normal goat serum diluted in 1× HBSS + 0.1% sucrose + 1.0% saponin for 20 min, and incubated overnight in a mouse monoclonal anti-Pax7 IgG1 primary antibody (dilution 1:100). The next day, cells were incubated in goat anti-mouse IgG1 biotin-SP-conjugated secondary antibody (dilution 1:1,000) for 60 min, incubated in streptavidin horseradish peroxidase conjugate for 60 min, amplified with Alexa Fluor 594 tyramide conjugate (dilution 1:200) for 20 min, and counterstained with 10 mM DAPI for 30 min. VectaShield antifade mounting media was added, and plates were stored at 4°C until imaging. Images were acquired using an Olympus IX-81 epifluorescent inverted microscope with the Olympus UPlanSApo 40×/0.95 NA long-working-distance objective and SlideBook 6.0 software. Scale bar = 20 μm.

7. Apply primary antibody: Add 75 μL (or appropriate volume) of the appropriate primary antibody to each section on slides 1–4.

a. Slides 1 and 2: Prepare 500 μL (working volume; 6 tissue sections) of Pax7 (1:100) + laminin (1:200) cocktail diluted in 2.5% NHS.

b. Slide 3: Prepare 300 μL (working volume; 3 tissue sections) of Pax7 (1:100) diluted in 2.5% NHS.

c. Slide 4: Prepare 300 μL (working volume; 3 tissue sections) of laminin (1:200) diluted in 2.5% NHS.

d. Slide 3 (Pax7 only) and slide 4 (laminin only) are used to determine the signal produced by background staining of the tissue. Thus, omitting the primary antibody should be included as a secondary control method.

e. To ensure the specificity of the primary antibody against Pax7, negative and positive controls must be included. For negative control, we used the C2C12 myoblast cell line with sp2/0-ag14 serum added to inhibit Pax7 protein expression (Figure 3). For positive control, we used the same C2C12 myoblast cell line with horse serum added to induce Pax7 protein expression (Figure 4).

f. Ensure the humidity chamber is damp and full of wet paper towels below the slides. Cover with the top of the humid chamber lid and carefully store at 4 °C overnight.

*Note: You can use parafilm to prevent air from entering the gap between the chamber and the lid. However, we had no problem with the primary antibodies evaporating by the next day.*

**Day 2:**

8. The next day, using a pipette, wash tissue sections on the slides four times × 5 min in 1× PBS.

9. Apply Pax7 secondary antibody to slides 1–3 (keep out of direct light from here on).

a. Add 75 μL (or appropriate volume) of secondary antibody specific to Pax7 (goat anti-mouse IgG1 biotin conjugated; 1:1,000 diluted in 2.5% NHS) and incubate for 1 h at RT in the dark (cover with aluminum foil).

*Note: Prior to preparing the dilution, quickly centrifuge the secondary antibody and take the solution from the top.*

b. Leave slide 4 in 1× PBS.

10. Using a pipette, wash tissue sections four times × 5 min in 1× PBS.

11. Apply laminin secondary antibody to slides 1, 2, and 4.

a. Add 75 μL (or an appropriate volume) of secondary antibody cocktail specific for laminin (goat anti-rabbit Alexa Fluor 488; 1:200) and streptavidin-HRP (1:100) diluted in 1× PBS and incubate for 1 h at RT in the dark (cover with aluminum foil).

*Note: In this case, streptavidin-HRP will be used to bind with tyramide conjugate in the next step to amplify the signal of Pax7.*

b. Leave slide 3 in 1× PBS.

12. Using a pipette, wash tissue sections three times × 5 min in 1× PBS.

13. Apply tyramide conjugate signal amplification.

*Note: Different protocols have been tested without tyramide conjugate (i.e., no amplification), but the labeling of satellite cells is suboptimal and not consistent. Specifically, there is low signal-to-noise from Pax7 labeling, sometimes there is no overlap between DAPI and Pax7 signals, and there is random nonspecific binding scattered all over the section.*

a. Add 75 μL (i.e., or appropriate volume) of Alexa Fluor 594 tyramide conjugate (1:200; diluted in 1× PBS) and incubate for 20 min at RT in the dark (cover with aluminum foil).

14. Using a pipette, wash tissue sections three times × 5 min in 1× PBS.

15. Apply DAPI counterstain.

a. Add 75 μL (or appropriate volume) of 10 mM DAPI and incubate for 30 min at RT. If short on time, it is acceptable to incubate in DAPI overnight. If there is overnight incubation, wash the tissue sections five times × 5 min in 1× PBS the next day.

16. Using a pipette, wash tissue sections three times × 5 min in 1× PBS.

17. Mounting:

a. Using a 20–200 μL pipette, cut off the bottom end of a 200 μL pipette tip to allow the suction of the viscous mounting media and prevent air bubbles.

b. Place 3–4 droplets (20–50 μL/slide) of the Vectashield antifade mounting media and carefully cover the slides with wide (22 × 50) coverslips, allowing the mounting medium to spread throughout the slide for 1–2 min.

*Note: Be very careful not to get any air bubbles covered over your sections and make sure the mounting media spreads similarly throughout your sections—not too much or too little.*

c. Carefully seal with nail polish by gently dabbing around the edges of the entire coverslip without moving it.

d. Store in a slide holder at 4°C in the dark until imaging. The sooner the image acquisition, the better. However, fluorescent labeling will last for several weeks if slides are stored properly (i.e., slides lying flat at 4 °C, in the dark).

**D. Confocal imaging acquisition**

This protocol is intended for SC visualization with a confocal microscope. Images were obtained on a Leica TCS SPE laser scanning confocal DM6-Q upright microscope equipped with four laser lines (405, 488, 561, and 635 nm) and an 8-bit spectral PMT detector with the Leica ACS APO 40×/1.15 NA oil objective and LAS X software version 3.5.7.23225. Z-stack images were acquired with a 0.44 μm step size for a total of 19.49 μm z-stack size (length), at 1,024 × 1,024 pixel resolution, 400 μs/pixel laser scan speed, and pinhole at 1 AU. Captured images were 275 × 275 μm in image size, 268.82 × 268.82 nm in pixel size, and 1.0 zoom factor. Additional instructions are as follows:

*Note: An epifluorescent (widefield) microscope can be utilized; however, this will sacrifice greater resolution and image quality. If using an epifluorescent microscope, the sample thickness should be sectioned thinner (i.e., 5–10 μm).*

1. Turn on the laser, microscope controller (i.e., if automated), and white light source.

2. Open the instrument software and load the slide.

3. Once the slide is loaded, move to a low-magnification objective (e.g., 10×/20×), change the port on the microscope from the computer to the objective lens, and adjust the z-position to focus your sample.

4. To best find and visualize the SCs within the sections, switch on the Texas red (TXR) 594 channel and search for a fluorescent signal on the focused section.

*Note: The channel used to probe for Pax7 will depend on the secondary antibody excitation/emission range. Alexa Fluor 594 excitation/emission range is 591/617 nm, which best corresponds to TXR 594.*

5. Once you find a small (i.e., 3–10 μm) oval/circular area of fluorescent signal, switch channels to DAPI channel (405 nm laser excitation) to visually confirm overlap between DAPI (nucleus) and TXR signal.

*Note: There will sometimes be tiny patches of high signal noise. Therefore, it is crucial to switch channels to DAPI to confirm signal overlapping. You can also switch to the green fluorescent protein (GFP) channel (488 nm excitation) to visually confirm the spatial context of the TXR signal; the TXR fluorescent signal should be completely adjacent to the GFP (i.e., laminin) signal.*

6. Once in position, change the low-magnification objective to a higher magnification (e.g., 40× or 63×) and/or proceed to the next step.

*Note: A higher magnification objective may require immersion oil. These objective lenses specifically require Type F immersion liquid (Leica Microsystems, Wetzlar, Germany, catalog number: 11513859). If using an oil immersion objective, it is important to keep your sections on the slide toward the middle so that the cover glass does not scrape the objective.*

7. On the software interface (see Video 1), add three sequential channels to set your filters: DAPI (405; for myonuclei), GFP or FITC (488; for Laminin), and TXR red (561; for SC nuclei)

a. For myonuclei stain (DAPI), set the percentage of laser intensity and the gain (voltage) of the PMT to obtain at least 60% of the relative fluorescent intensity from the histogram, assign a pseudo-color (e.g., blue) to this signal, select the emission for DAPI, and set emission range to 430–480 nm.

b. For laminin (GFP or FITC), set the percentage of laser intensity and the gain (voltage) of the PMT, select Alexa Fluor 488 emission (i.e., or appropriate secondary antibody used), assign a pseudo-color (e.g., green) to the signal, and set emission range to 500–540 nm.

c. For Pax7 (TXR red), set the percentage of laser intensity and the gain (voltage) of the PMT, select Alexa Fluor 594 emission (i.e., or appropriate secondary antibody used), assign a pseudo-color (e.g., red) to the signal, and set emission range to 600–640 nm.

*Note: Modify the excitation intensity, gain, and emission range histogram for channels depending on how much contrast is displayed in the image preview. In our experience, we have had to modify these aspects depending on the different reagents utilized and section thickness. For example, if the brightness is too extreme (saturated), reduce the laser intensity first and then the gain depending on the fluorescence intensity shown in the histogram.*

**Video 1.**
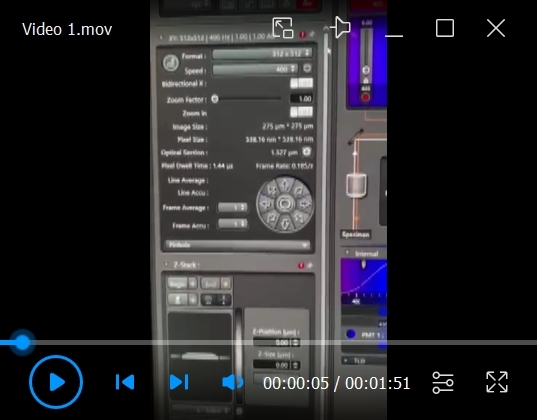
Setting up channels in the LAS X software operating the Leica SPE laser scanning confocal microscope

8. Select a low-pixel-resolution format (e.g., 512 × 512) and live mode to select the region of interest.

9. Determine the z-stack size (see Video 2) by focusing up/down and selecting the range from the sample depth. Carefully adjust the z-position of the objective to where there is no longer any signal in the one direction (e.g., objective further away from the sample) and click set/start position on the computer. Then, move the z-position toward the opposite direction (e.g., objective closer toward the samples) to where there is no longer any signal, and click the end position on the software to acquire the total volume.

**Video 2.**
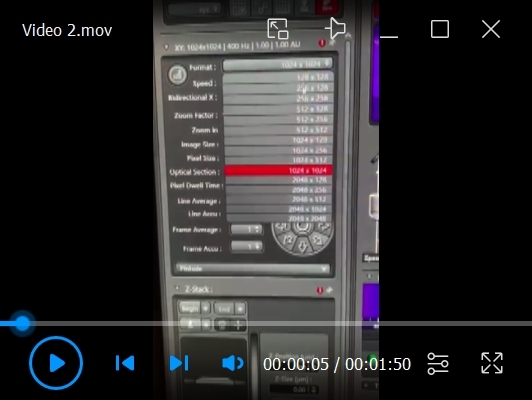
Setting the z-stack in the LAS X software operating the Leica SPE laser scanning confocal microscope

10. Change pixel resolution format to 1,024 × 1,024 and begin/click image acquisition.

a. While executing, record the z-stack size, z-step size, laser scan speed, zoom factor, image size, pixel size, optical section, and pinhole size.

*Note: For confirmation purposes, all SCs should be adjacent to the laminin signal and overlapping Pax7 and DAPI signals in the center of the nuclei.*

**E. Image post-processing using 3D Viewer software (LAS X)**

1. Once the image is acquired, observe the image using 3D viewer.

2. Change the background to black and select overlay under the *Volume* tab.

3. Begin modifying the histogram range (“...” icon) under the *Volume* tab of each channel (DAPI, Pax7, and laminin) to optimize image resolution and contrast. Take note of each image’s channel histogram ranges.

*Note: Each image should have a similar range (e.g., low: 3; high: 250), although the range values will differ depending on what objective was used. Typically, place the values on the histogram hugging just outside the bulk of the signal so that details of the image are not lost. Gamma must remain at 1.0.*

4. Set the intensity to 100 and modify as needed depending on the brightness of the raw image. However, intensity values should also be similar across images.

5. Using the left (rotation) and right (x, y movement) clicker and rubber wheel (zoom) of the computer mouse, move through the image to the desired location and collect zoom percentage information.

6. Set scale bar.

*Note: We set the scale bar to Times New Roman, font size 26. This is optional, but you can add a frame or axis scaling to visualize the image.*

7. Once image quality is optimized, click *save image as* and save as TIFF with a standard or custom frame size (e.g. 1,024 × 1,024).

*Note: The instructions above are for 3D visualization of the channels combined (overlay). In 3D viewer, you can also separate the channels into individual quadrants with one quadrant as an overlay if you wish to visualize the channels separately.*

## Validation of protocol

**Figure 5.**
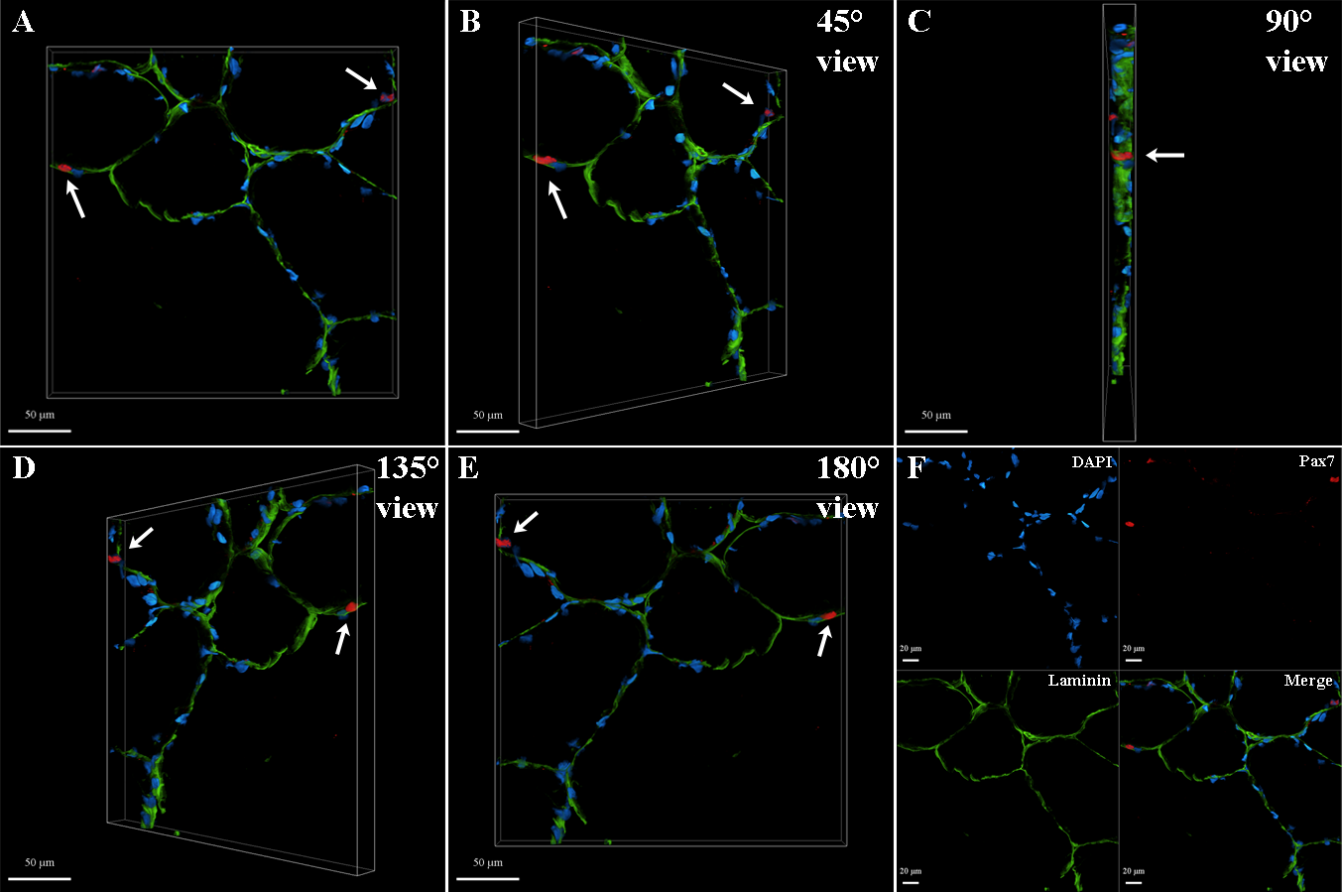
Representative three-dimensional (3D) images in rotating sequence, identifying human satellite cell nuclei (arrows) from vastus lateralis skeletal muscle tissue immunolabeled with a monoclonal anti-Pax7 primary antibody. Z-stack images showing satellite cell nuclei through Pax7^+^ (red; white arrows) labeling overlapped with DAPI (blue) along the membrane of the muscle fiber via laminin labeling (green). Red fluorescent signal was excited at 561 nm with 40% laser power intensity and 700 V PMT gain; green fluorescent signal was excited at 488 nm with 8% laser power intensity and 700 V PMT gain; blue fluorescent signal was excited at 405 nm with 6% laser power intensity and 700 V PMT gain. Fluorescence emission ranges for red, green, and blue were 600–640 nm, 505–540 nm, and 430–480 nm, respectively. Image post-processing was carried out using the Leica LAS X 3D Viewer software, where brightness intensity was set to 200, and channel signal histograms (minimum-maximum) were set for better display as 10–65, 5–165, and 20–250 for Pax7, laminin, and DAPI, respectively. (A) Front image zoom percentage at 110; (B) Rotated (45°) image zoom percentage at 110; (C) Rotated (90°) image zoom percentage at 110; (D) Rotated (135°) image zoom percentage at 110; (E) Backside (180°) image zoom percentage at 110; (F) Channels and overlay, image zoom percentage at 70. Scale bar = 50 μm (A–E) and 20 μm (F).

Following the detailed workflow above, SC nuclei can be visualized in a three-dimensional view to observe morphological characteristics (Figure 4) and later perform downstream quantification analyses. Figure 5 was generated from a 27-year-old, well-trained male subject days after an intense lower body strength training session. Validation of these results is based on 10 replicates, all showing the spatial context of the fluorescent labeling (i.e., satellite cell nuclei on the border of the muscle fiber immunolabeled using laminin) and multichannel signal overlap of Pax7 and DAPI in three-dimensional volume.

## General notes and troubleshooting

1. We acknowledge that this protocol involves many steps and can be overwhelming for a beginner or a student. However, we have taken care to provide detailed step-by-step instructions, including necessary methodological clarifications and troubleshooting, to enhance the reproducibility for a wide range of individual skill levels.

2. This protocol has also worked on thinner sections (i.e., 10 μm) but that would be ill-advised if the goal is to observe a greater depth volume. Therefore, sectioning at 20 μm or more will be beneficial for visualizing SCs in three-dimensional space.

3. Originally, we obtained very inconsistent results for the Pax7 labeling by using cocktails of both primary and secondary antibodies. After several troubleshooting experiments, we confirmed that adding the Pax7 secondary antibody first and then the laminin secondary antibody ensures appropriate binding for both targets.

4. We strongly recommend Pax7 amplification using the tyramide conjugate. This product can also come in an Alexa Fluor^TM^ 594 Tyramide SuperBoost^TM^ kit, goat anti-mouse IgG (tyramide reagent, streptavidin-horseradish peroxidase conjugate; Thermo Fisher Scientific, catalog number: B40942). We have tried the immunofluorescence protocol without Pax7 amplification following other methods, but results were not optimal. We have tried with varying buffers, antibodies, antibody dilutions, and incubation periods over 15 times. If amplification is not utilized, expect to see a nonspecific background of Pax7 signal outside the nuclei. We have used wheat germ agglutinin (Alexa Fluor 488 conjugate; dilution 1:200) to stain glycosaminoglycans within the basal lamina instead of laminin to label the sarcolemma (plasma membrane) of the fibers. Both work equally well.

5. The protocol described here is for human skeletal muscle cross-sections; however, the method for 3D visualization of satellite cells may work on longitudinal sections, other tissue sections (e.g., rodents), and/or other types of microscopes.

6. We recommend sections adhering to slides and do not recommend free-floating immunohistochemistry after sections are generated due to poor Pax7 labeling. Muscle fibers are very delicate; muscle samples can easily break apart if sections are floating.

7. A spinning disk confocal microscope has also been utilized. Although the results generated with a spinning disk confocal microscope were adequate, a laser scanning confocal microscope was preferred.
